# The Influence of Meteorology on the Spread of Influenza: Survival Analysis of an Equine Influenza (A/H3N8) Outbreak

**DOI:** 10.1371/journal.pone.0035284

**Published:** 2012-04-20

**Authors:** Simon M. Firestone, Naomi Cogger, Michael P. Ward, Jenny-Ann L. M. L. Toribio, Barbara J. Moloney, Navneet K. Dhand

**Affiliations:** 1 Faculty of Veterinary Science, The University of Sydney, Camden, New South Wales, Australia; 2 The EpiCentre, Institute of Veterinary Animal and Biomedical Sciences, Massey University, Palmerston North, New Zealand; 3 New South Wales Government Department of Primary Industries, Principal Department of Trade and Investment, Regional Infrastructure and Services, Orange, New South Wales, Australia; National University of Singapore, Singapore

## Abstract

The influences of relative humidity and ambient temperature on the transmission of influenza A viruses have recently been established under controlled laboratory conditions. The interplay of meteorological factors during an actual influenza epidemic is less clear, and research into the contribution of wind to epidemic spread is scarce. By applying geostatistics and survival analysis to data from a large outbreak of equine influenza (A/H3N8), we quantified the association between hazard of infection and air temperature, relative humidity, rainfall, and wind velocity, whilst controlling for premises-level covariates. The pattern of disease spread in space and time was described using extraction mapping and instantaneous hazard curves. Meteorological conditions at each premises location were estimated by kriging daily meteorological data and analysed as time-lagged time-varying predictors using generalised Cox regression. Meteorological covariates time-lagged by three days were strongly associated with hazard of influenza infection, corresponding closely with the incubation period of equine influenza. Hazard of equine influenza infection was higher when relative humidity was <60% and lowest on days when daily maximum air temperature was 20–25°C. Wind speeds >30 km hour^−1^ from the direction of nearby infected premises were associated with increased hazard of infection. Through combining detailed influenza outbreak and meteorological data, we provide empirical evidence for the underlying environmental mechanisms that influenced the local spread of an outbreak of influenza A. Our analysis supports, and extends, the findings of studies into influenza A transmission conducted under laboratory conditions. The relationships described are of direct importance for managing disease risk during influenza outbreaks in horses, and more generally, advance our understanding of the transmission of influenza A viruses under field conditions.

## Introduction

Influenza A viruses are enveloped RNA viruses of the family *Orthomyxoviridae*, and a major cause of morbidity and mortality in both humans and livestock, worldwide [1], [2], [3]. Spread may be via direct contact, over short distances on large ‘cough’ droplets (diameter >10 µm), over longer distances in aerosols of small droplet nuclei (diameter <10 µm) and on fomites [4], [5]. Meteorological variables such as air temperature, relative humidity, rainfall and wind have been suggested as important drivers of the spread and seasonality of influenza in both human [5], [6], [7], [8] and animal populations [9]. Recently, Lowen et al. described, under laboratory conditions, how relative humidity and ambient temperature combine to influence the transmission of both seasonal (A/H3N2) and pandemic (A/H1N1) human influenza A [6], [8], [10]. The effects of several other environmental variables (soil pH, sunlight and surface permeability) on the survivability of influenza A viruses were established in earlier laboratory-based experimentation [11], [12]. Analyses of the contribution of wind to the spread of epidemics of influenza, and indeed other infectious diseases, are more limited. Most studies present either circumstantial evidence that the mean direction of epidemic spread coincides with prevailing wind conditions at the time of an outbreak [13], [14], analyses of data aggregated to a low temporal or spatial resolution [15], [16], or associate spread from a small number of sources with atmospheric dispersal modelling outputs [17]. Such research must also overcome the added complexity of movement of individuals within the population at risk.

In their animal model of human influenza A transmission, Lowen et.al. have shown that dry cool conditions (low relative humidity and cold ambient temperatures) increase the spread of influenza [6]. They suggest that this mechanism is mediated by a complex interaction that affects the survivability of both aerosol droplet nuclei and virus particles. A detailed analysis, at high spatial and temporal resolution, comparing actual influenza outbreak data with concurrent meteorological data is required to validate and provide context to their model outside of controlled laboratory environments, thus furthering our understanding of how meteorological factors truly influence influenza spread. Outbreaks of disease in animal populations present a unique opportunity to study such effects, ‘in the field’. Research on detailed animal outbreak datasets has several distinct advantages over comparable research on public health influenza data [15], [18], [19], [20]. Firstly, human populations move about on a daily basis (albeit with some regularity). Implementing a complete human movement standstill (‘a 24 hour curfew’) to control and contain an outbreak is considered an extraordinary and perhaps unfeasible social distancing measure, reserved for the most severe of human influenza pandemics [21]. Conversely, the movements of farm animal populations (such as horses, cattle and sheep) are mostly confined to within single premises, and in the event of an emergency animal disease outbreak, a complete movement ban is often the first control measure to be implemented [22]. Furthermore, ethical concerns (namely privacy) may constrain the research of human outbreak data, limiting the amount of detailed information that can be collated on the movement of individual people. Given that certain human and animal sub-types of influenza A share generally similar modes and patterns of transmission [5], research that utilises detailed animal outbreak datasets has the potential to inform our understanding of the complex mechanisms that influence human influenza A spread and seasonality. The 2007 outbreak of equine influenza in Australia presented an excellent opportunity to study the effects of meteorology on the spread of an influenza A virus as it infected a mostly immunologically naive population, spatially confined (in paddocks).

Equine influenza virus (A/H3N8) is a highly contagious cause of low mortality, high morbidity respiratory disease capable of infecting all members of the horse family (*Equidae*). It is considered endemic to equine populations across most of the world [2]. The disease is similar in many clinical and epidemiological respects to seasonal human influenza A, and major outbreaks have occurred when novel strains of equine influenza have gained entry into highly susceptible equine populations [2]. The typical incubation period of equine influenza is 1–3 days [23], [24], [25], however delayed onset of clinical signs of up to 5 days has been observed after low dose aerosol exposure [26]. In 2007, following a breach in the quarantine of infected imported horses [27], Australia experienced its first ever outbreak of equine influenza. Less than 900 horses are imported annually into Australia from countries that vaccinate for equine influenza [27], therefore almost the entire horse population was susceptible at the start of this outbreak. Over the course of 4 months, nearly 70 000 horses were infected, on over 9 000 premises in two Australian States—New South Wales (NSW) and Queensland (QLD) [27]. Timely and complete implementation of a horse movement ban has been widely credited as the most effective of the control measures that facilitated the rapid eradication of this disease from the Australian horse population [27]. Although vaccination was used to eradicate the disease, its implementation only commenced 6 weeks into the outbreak, well after the peak of reported daily infections [28]. Vaccination was initially restricted to disease containment zones and the protection of high value horses [28].

Contact-tracing early in the 2007 outbreak revealed that the disease initially spread through a network of equestrian events, linked by the movement of infected horses prior to detection of the outbreak, producing clusters of infected premises in widespread locations [27], [29]. Epidemiological investigations noted rare instances of presumed windborne spread over short ranges (≤1.5 km, and rarely up to 5 km) based on failure to identify other potential means of transmission (i.e. close contact or on fomites) [30]. Previous epidemiological analyses of this outbreak have investigated the spatial and network components of early spread [29], [30], [31], [32], and premises-level risk factors for disease spread such as compliance with advised biosecurity measures [33]. Two further analyses have specifically investigated environmental factors that might have influenced the spread of this outbreak [13], [34]. In one cluster of 437 infected premises, a relationship was observed between prevailing wind conditions and the global direction of spread [13].

In this paper we present a comprehensive analysis of the influence of meteorological variables on time to infection based on an influenza A virus outbreak dataset. This spatio-temporal analysis aims to identify and quantify the association between four meteorological variables (air temperature, relative humidity, rainfall, wind velocity) and time to infection in the largest cluster of the 2007 equine influenza (A/H3N8) outbreak in Australia. We are unaware of any previously published analysis that combines such a large and spatio-temporally detailed influenza outbreak dataset with concurrent daily meteorological data, to allow meaningful estimation of the contribution of such factors in the spread of an influenza A outbreak.

## Materials and Methods

### The equine influenza dataset

The state government of New South Wales provided contact-tracing and laboratory testing data on all horses investigated during the 2007 outbreak. This dataset was collected at the level of individual horses and aggregated to the premises level for analysis. Study designs that use groups as the unit of interest (such as herds or flocks) rather than individuals, are common in veterinary epidemiological research [35]. Premises attribute records included address, geocoded coordinates (based on premises centroid), number of horses, date of onset of clinical signs in the first horse affected (‘onset date’), vaccination status and date of vaccination. Premises were defined as infected (IP) if they held horses that had been observed with the classical clinical signs of equine influenza (cough, elevated temperature, nasal discharge and lethargy). This status was confirmed by laboratory testing based on real-time reverse transcription polymerase chain reaction assay [36], however, around the peak of the outbreak not all horses were tested due to resource constraints [30]. Contact-tracing records included the date of the movement, the addresses and unique identifiers for the origin and destination premises between which horses were moved prior to the horse movement ban.

### Study extent: cluster delineation

**Figure 1:**
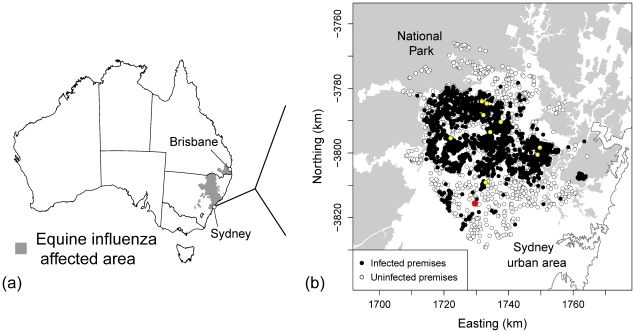
Map of Australia showing the area affected by the 2007 outbreak of equine influenza and the study extent. (a) From August–December 2007, around 70,000 horses were infected on over 9000 horse premises in two Australian States. (b) This study focused on the largest cluster (n = 3624 horse premises), northwest of Sydney, as defined by a 15 km buffer around the nine earliest infected premises (depicted in yellow) that were contact-traced to events where disease transmission was known to have occurred in the first week of the outbreak. Clinical signs were first observed on 17 August 2007 in a horse in quarantine at Eastern Creek Quarantine Station (red closed circle). The cluster is surrounded by national parks and Sydney urban areas. (For interpretation of the references to colour in this text, the reader is referred to the web version of the article.).

There was a single ‘index’ for the 2007 outbreak of equine influenza in Australia: an equestrian event located 160 km north of Sydney, at which transmission was known to have occurred. This analysis focused on local spread within the single largest cluster of the outbreak, centred 60 km northwest of Sydney's city centre (Figure 1). To maintain a computationally tractable dataset, premises were selected for inclusion in the study (from the equine influenza dataset) if their centroid was within 15 km of nine contact-traced ‘source’ premises. All nine contact-traced premises were identified (based on an earlier likelihood-based analysis [32]) to have been infected in the first week of the outbreak following the movement of infected horses from the ‘index’. The 15 km buffer used to delineate the cluster was selected based on a previous analysis in which we identified that 98% of premises infected in the first month of the outbreak were within this distance of a contact-traced ‘source’ premises [29]. The ‘Northwest Sydney’ cluster studied was approximately 65 km in diameter, bounded to the North and West by national parks (where horses are prohibited) and to the South and East by metropolitan Sydney.

### Exploratory spatial and temporal analyses

The dataset was imported into the R statistical package version 2.13.0 [37], and an epidemic curve constructed as the count of infected premises reported per day. The spatial coordinates of each premises were converted to the Australian Albers conic equal-area projection which is based on the Geocentric Datum of Australia 1994 (www.ga.gov.au/geodesy/datums/gda.jsp). Extraction mapping was used to investigate the spatial pattern of risk of infection over time. To identify areas of elevated risk, relative risk surfaces with upper 95% tolerance contours were estimated as the Gaussian-smoothed kernel density surface of infected horse premises divided by the surface of the population of horse premises at risk in 4-week time periods. A spatially adaptive variable smoothing parameter was used to prepare the relative risk surfaces [38], with edge effect correction, implemented in R with the ‘sparr’ library [39]. The amount of smoothing (bandwidth) applied varied across the study extent in inverse proportion to the population at risk in each time period. To test for directional spread, the mean geographic centre of the outbreak was estimated by week as the mean of the coordinates of the infected premises with dates of onset in each week of the outbreak [40].

### Survival analysis

We applied semi-parametric Cox regression modelling to estimate the association between potential risk factors and the times to infection of individual premises. A geodatabase was compiled in Microsoft Access 2007 (Microsoft Corporation, Redmond, WA, USA) to maintain all premises and meteorological data, with spatial covariates added using ArcMap 9.3 (ESRI, Redlands, CA, USA). The dataset was structured into a daily ‘counting process’ formulation to enable investigation of the effects of time-varying predictors [41], in this case time-lagged premises-level meteorological variables. In this formulation, each premises contributes one observation for every day that it is at risk (until either clinical signs are observed in horses on the premises, or the end of the study period). See Supporting Information S1 for a sample of the survival dataset used in this analysis. Time-varying covariates and the counting process formulation were arranged using the R statistical package.

In the counting process generalisation of the Cox proportional hazards model, the hazard function depends on time in ways other than only through the baseline hazard function [42]. The proportional hazards assumption does not apply, allowing for inclusion of time-dependent covariates [43]. Each subject contributes one observation for every day that it is at risk and each observation contains covariates for the subject at each time point of observation and a start and stop time denoting the interval of risk, *i.e.* (start, stop] [44]. This enables covariate values for individual subjects to either be time-invariant or to change with time, and to be incorporated into a generalised Cox regression model [41] of the form:
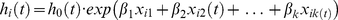
(1)where *h_i_*(*t*) is the hazard that an individual, *i*, from the population yet to experience an event, will experience the event at time *t*; *h_0_*(*t*) is the baseline hazard at time *t*; *β1* and *β2* are the regression coefficients for the time-invariant, *x_i1_*, and time-dependent covariates, *x_i2_*_(*t*)_, respectively. The partial likelihood specification for the counting process Cox regression model is described in detail by Anderson and Gill (1982), and is estimated including a term for each unique event time, summing over those observations that are still at risk at each actual event time. As there is no overlap in intervals of risk in the set of observations for each subject, the likelihood never involves more than one observation for a subject [44].

Network and spatial spread in the early outbreak period (the first 14 days of this outbreak) is described in detail elsewhere [32]. To focus this analysis on the meteorological factors associated with local spread, we excluded any premises that may have been infected in the first 10 days of the outbreak, before the complete implementation of horse movement bans (i.e. any premises with an onset date in the first 14 days of the outbreak), setting the origin of the survival analysis at 30 August 2007. This period ends one typical incubation period (3 days) after movement bans were implemented, with an additional 1 day error margin for delay in observation and reporting [33]. All premises that remained uninfected on the 131^st^ day of the outbreak (25 December 2007, the reported date of onset of the last known infected premises) were right censored on this date.

#### Explanatory variables

Explanatory covariates tested for associations with the time to infection of premises in the Northwest Sydney cluster are listed in Table 1. Premises boundaries were extracted from cadastral data provided by the NSW Government Department of Finance and Services. These boundaries were used to generate a continuous variable representing the length of fence that each horse premises shared with any contiguous horse premises in the equine influenza dataset. Premises elevation was extracted from a digital elevation model of Australia [45], which is a grid of ground level elevation covering the whole of Australia with a grid spacing of approximately 250 metres, as the mean of all grid cells needed to cover a premises. Distance to the nearest main road was calculated from the premises boundary using vector data of road Classes 1–3 (freeways, highways, primary and arterial roads) [46]. Human population density, within approximately 1 km of the premises centroid, was estimated based on high resolution gridded population data from 2005 [47], adjusted by 3% for population growth between 2005 and 2007 [48].

**Table 1:** Explanatory variables analysed for associations with time to infection of premises in the largest cluster, northwest of Sydney, during the 2007 equine influenza outbreak in Australia.

Variable group	Variable name	Variables (Units)
***Meteorological***	RAIN *t_−1_,t_−2,_…,t_−5_*	Rainfall (mm day^−1^)^a^
***covariates***	RH_9AM *t_−1_,t_−2,_…,t_−5_*	Relative humidity (%) measured daily at 9am^a^
***(time-lagged)***	RH_3PM *t_−1_,t_−2,_…,t_−5_*	Relative humidity (%) measured daily at 3pm^a^
	TEMP_MAX *t_−1_,t_−2,_…,t_−5_*	Maximum daily air temperature (°C)^a^
	TEMP_MIN *t_−1_,t_−2,_…,t_−5_*	Minimum daily air temperature (°C)^a^
	WIND_SPD_undir_ *t_−1_,t_−2,_…,t_−5_*	Maximum daily wind speed – undirected (km hour^−1^)^a^^,^ b
	WIND_SPD_dir(*k*)_ *t_−1_,t_−2_,…,t_−5_*	Maximum daily wind speed – directed (km hour^−1^)^a^^,^ b
***Premises***	AREA	Area (acres)
***attributes***	HORSE_DENSITY	Horse density (horses acre^−1^)
	HORSES_NUMBER	Number of horses
	SHARED_FENCE	Length of shared fence with other horse premises (m)
	VACC	Vaccination status (1 = Yes, 0 = No)^a^
	VACC_DAYS	Days since vaccination^a^
***Spatial***	ELEV	Elevation (m)
***covariates***	HUMAN_DENS	Human population density within approximately 1 km of the premises (people km^−2^)
	ROAD_DIST	Distance to nearest main road (km)^c^

a Time-changing covariate.

b Maximum daily wind speed was either based on wind from all directions (‘undirected’) or wind only from within 45° arcs centred on the direction of the *k* nearest infected premises for *k* = 1,2,3 (see Figure 2 for details) assuming that premises were infectious for 14 days and one of the nearest *k* infective premises was the source of infection.

c Main roads include freeways, highways, primary and arterial roads (Classes 1–3).

#### Estimation of meteorological time-varying predictors

Hourly wind velocity data (wind direction and speed) and daily data for five other meteorological variables (rainfall, minimum and maximum daily air temperature, and relative humidity measured at 9 am and 3 pm) were obtained from 132 weather stations. All of these weather stations were operated by the Australian Bureau of Meteorology during the study period, and were located either within the cluster or within 20 km of the cluster boundary. Most stations reported only daily rainfall measurements. Ordinary kriging [49] was used to interpolate daily values at each individual premises location for the meteorological time-varying predictors: maximum wind speed (km hour^−1^), rainfall (mm), maximum and minimum surface air temperature (°C), and relative humidity (%, measured at 9 am and 3 pm). Each time-varying meteorological covariate was then time-lagged by 1–5 days to encompass the full range of incubation periods observed in experimental infection studies [26].

Kriging is a geostatistical smoothing technique that involves modelling the underlying spatial dependency (autocorrelation) in spatially continuous data based on a covariance function (Figure 2a) [49]. For each observation point (hour or day), for each meteorological variable, a binned isotropic empirical variogram was plotted that represented covariance (as semivariance) up until half of the maximum pairwise distance between any two weather stations contributing data at that time point, with bin widths (*h*) of approximately 10% of the average distance between weather stations [50]. A stationary exponential variogram model was then fit to the empirical variogram, using iterative least squares regression, and parameter estimates used to interpolate values at each premises location [49].

**Figure 2:**
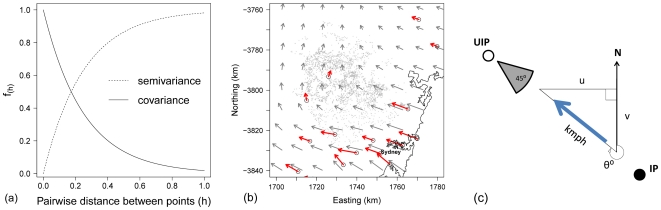
The estimation of premises-level wind speed covariates in a survival analysis of time to infection in the largest cluster of the 2007 outbreak of equine influenza in Australia. (a) Exponential covariance function (with practical range = 0.25) and its related semivariance function. (b) Hourly wind velocity data from sixteen automated weather stations (open circles) within a 20 km buffer of the cluster's boundary were converted into their East-to-West (‘u’) and North-to-South (‘v’) components, and smoothed using kriging to predict hourly wind speed and direction at each premises (small grey dots). (c) For each premises on each day prior to infection or censoring, the (‘directed’) maximum wind speed originating from within 45( arcs centred on the direction of the nearest 1–3 infected premises was estimated for time lags of 1–5 days.

#### Generation of wind speed covariates

Hourly wind velocity data were available from sixteen of the weather stations, automatically measured on masts at 10(metres above the earth's surface. These wind data were supplied in a polar coordinate structure, comprising the average direction of origin of the wind (in degrees from true north) and the maximum wind speed (in kilometres hour(1), measured over the 10(minutes leading up to the observation time. To avoid the issue of northerly bearings being split at true north (i.e. true bearings of 1( and 359( seeming distant when they are only 2( apart), prior to variography and kriging, the wind velocity data was converted into a Cartesian coordinate system—defined by two components (Figure 2b,c): “u" representing the East-to-West component of the wind velocity, and “v" representing the North-to-South component [51]. A negative value for the “u" component therefore represents a wind from one of the westerly bearings (i.e. NW, W or SW).

Kriging was then conducted on the two wind velocity vector components [51]. Hourly wind velocity vectors were interpolated for each premises and back-transformed into the original polar coordinates (direction of wind origin and maximum wind speed).

Two approaches were taken to aggregate the hourly wind velocity vectors for each premises into daily maximum wind speed covariates. First, to test the hypothesis that increased wind speed from any direction was associated with increased hazard of infection we generated ‘undirected’ maximum daily wind speed covariates (‘WIND_SPD_undir_’) without making any directional assumptions, taking the maximum of all hourly wind speed estimates for each premises on each day.

Next, to explore the directionality of wind exposure risk we generated ‘directed’ maximum daily wind speed covariates (‘WIND_SPD_dir_’) based only on wind coming from within the direction of the nearest *k* infected premises (for *k* = 1,2,3) by selecting wind from within 45° arcs centred on the bearing of the nearest *k* infected premises to each premises on each day. For each premises, on each day of observation, we identified the nearest three infected premises from amongst those infected premises that had a date of onset (of clinical signs in the first horse infected on the premises) within the previous 14 days. Though it is known that individual unvaccinated horses remain infectious for up to 7 days [2], [52], the duration of infectivity may vary on multi-horse premises because of differences in contact rates between individual horses, and individual variability in susceptibility, latency and virus shedding. To infer which premises were holding infectious horses at each time point we assumed that the period of infectivity was 14 days for all premises based on case reports from horse premises of a range of sizes [53], [54], [55], intra-herd simulation modelling [28] and that almost the entire population was immunologically naive to equine influenza at the start of the outbreak.

Finally, these time-varying predictors were lagged by 1–5 days to serve as proxies for wind within the range of incubation periods that have been observed for equine influenza, producing 20 time-lagged explanatory covariates: ‘WIND_SPD_undir_ *t_−1_*, *t_−2_*, …, *t_−5_*’ and ‘WIND_SPD_dir(*k*)_ *t_−1_*, *t_−2_*, …, *t_−5_*’, for *k* = 1,2,3.

### Univariable analysis

Instantaneous hazard curves were constructed for each time-invariant covariate with the ‘epiR’ library in R [56], categorising continuous variables into quartiles. The instantaneous hazard rate, *h*(*t*), is the rate at time *t*, that a randomly-selected individual from the population yet to experience an event, experiences the event at time *t* [43], and is mathematically defined as:
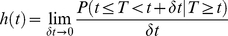
(2)where *T* is the time that an event is experienced. In this study, the unit of interest was the horse premises, and events were defined as the infection of horses with equine influenza virus on a previously uninfected premises.

Univariable Cox models were then constructed and the statistical strength of the association between each variable (categorical or continuous) and the outcome assessed using likelihood ratio tests [35]. The linearity of the relationship between log hazard of infection and each continuous variable was assessed graphically using restricted cubic splines [57] with knots spaced at quintiles in the data. To differentiate linear and nonlinear component terms, partial likelihood ratio tests were conducted comparing a model containing all spline terms to a nested model containing only a single linear term [57]. If a highly non-linear relationship was detected, the spline of the continuous variable was retained for multivariable analysis. All continuous covariates were tested for collinearity in pairs by calculating Spearman's rank correlation coefficient (*ρ*). Intrinsic temporal autocorrelation was expected amongst certain groups of time-lagged time-varying meteorological predictors, such as: ‘TEMP_MIN *t_−1_*’with ‘TEMP_MIN *t_−2_*, …, *t_−5_*’ and ‘RH_3PM *t_−1_*’ with ‘RH_9AM *t_−1_*, *t_−2_*, …, *t_−5_*’. From amongst any pair of highly correlated (*ρ*>|0.70|) time-invariant covariates, and from amongst intrinsically temporally autocorrelated groups of time-varying predictors, only the variable with the strongest statistical association with the outcome was retained for further analysis [58].

### Multivariable analysis

All remaining variables (unconditionally statistically associated with the log hazard of infection at *P*-value<0.25) were entered into a generalised ‘counting process’ Cox regression model [41]. Each eligible candidate variable was then individually tested by excluding it from the maximal model and conducting likelihood ratio tests, eliminating any variables with *P*-value≥0.10. To assess confounding, all eliminated variables were individually added back into the model, retaining any terms that resulted in a >20% change in any regression coefficient. The time-varying predictor representing vaccination status was forced into all multivariable models as it was considered *a priori* to confound disease spread. The linearity of the relationship between the outcome and each continuous variable still included in the model was assessed again, using restricted cubic splines [57]. Finally, tests were conducted for all two-way interactions of terms in the preliminary main effects model.

Goodness of fit of the final model was assessed using ‘Martingale’ residuals. The influence of every individual observation was tested by omitting it and observing for change in the regression coefficients [59]. To test for spatial dependency (autocorrelation) we examined the spatial structure of the residuals of the final model by mapping normalised martingale residuals (‘deviance residuals’) and plotting an empirical semivariogram [60].

## Results

### Exploratory spatial and temporal analysis

The Northwest Sydney cluster of the 2007 equine influenza outbreak in Australia contained 3624 horse premises, of which 1922 were reported to be infected during the 131 day outbreak (cumulative incidence = 53.0%, 95% CI: 51.4, 54.7%).

**Figure 3:**
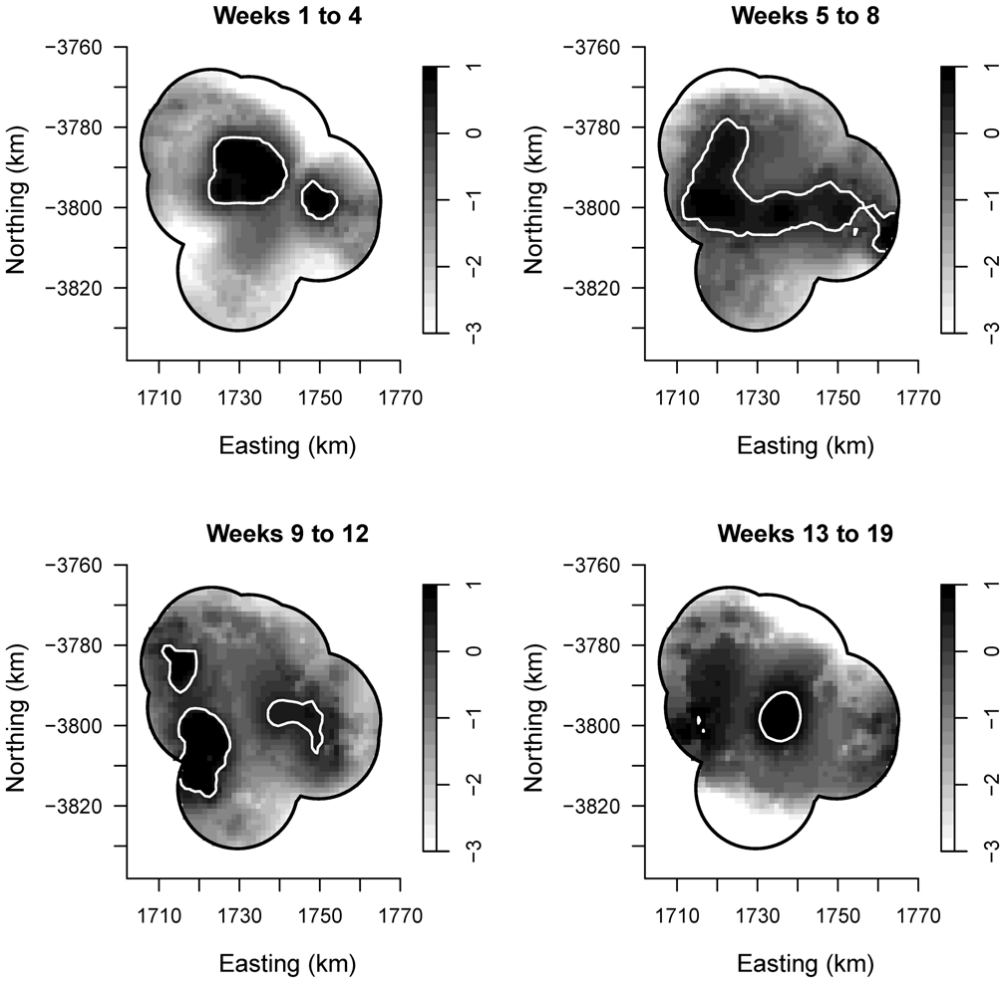
Spatial spread of equine influenza in the largest cluster of the 2007 outbreak in Australia. Surfaces of log relative risk were estimated in 4-week intervals using adaptive kernel estimation, with upper 95% tolerance contours (solid white lines). With this method the amount of smoothing (bandwidth) is inversely proportional to the density of the population at risk.

Surfaces of spatial relative risk by four week period are included as Figure 3. In the first 4 weeks of the outbreak there were two areas of elevated spatial risk localised around the nine source premises for this cluster. Over the next 4 weeks, the two areas of elevated risk coalesced and expanded. Between weeks 9 to 12, the areas of spatial risk dissipated into several smaller pockets of infection. Over the remainder of the outbreak, the spatial risk faded out in isolated pockets of infection.

The mean centre of the outbreak did not move predominantly in any single direction over the study period, moving Northwest at 3.0 km week^−1^ in the first 4 weeks, then Southwest at 3.9 km week^−1^ for 4 weeks, before moving back to the East at 4.1 km week^−1^ whilst the epidemic faded out.

### Survival analysis

**Figure 4:**
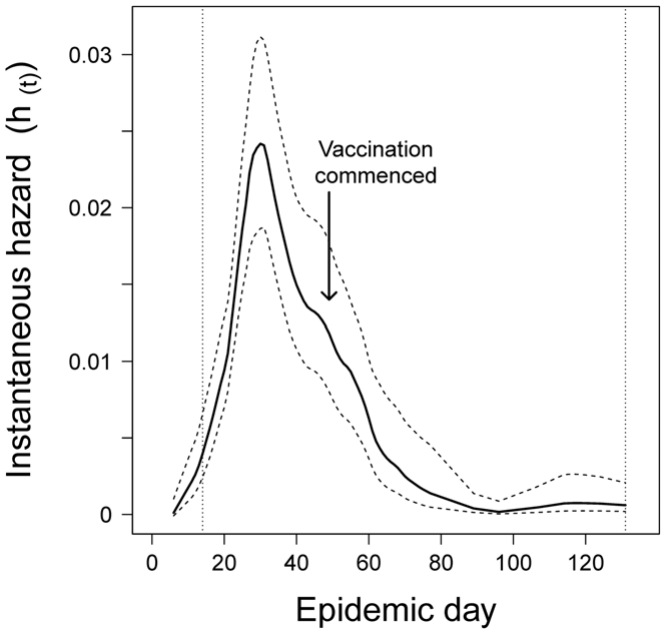
Smoothed instantaneous hazard of infection in the largest cluster of the 2007 outbreak of equine influenza in Australia. Horse movement standstills were implemented from day 10, and vaccination commenced in this cluster on day 49. Dashed lines represent 95% confidence intervals, and dotted vertical reference lines denote the survival analysis study period (between days 14 and 131 of the outbreak).

The complete survival dataset included 3153 premises containing 1727 events (infections) during the study period. Data on 57 infected horse premises were excluded because their onset dates occurred in the first 14 days of the outbreak (a period when they could possibly have been infected by the movement of infected horses rather than by local spatial spread). Sixty-seven infected premises were missing a date of onset, and 347 premises (71 infected and 276 uninfected premises) were missing data on their number of horses. Once data on these premises (which were evenly distributed across the study extent) had been excluded, data on all variables were complete. The median survival time, the point at which half of the premises in this cluster were infected, was day 55 of this outbreak (95% CI : 52, 61). The instantaneous hazard, the proportion of infections per day in the population surviving uninfected until that day, peaked on day 28 (Figure 4); 92 premises were reported to be infected on this day.

### Univariable analysis

#### Meteorological covariates and hazard of infection

Most horse premises were relatively close to a weather station, with the mean distance to the nearest weather station reporting wind data being 11.7 km (SD = 5.4 km, maximum = 27.4 km). For all meteorological data, there was a paucity of weather stations in the Northwest corner of the study extent (because this region is bordered by a national park).

Daily rainfall data were available from 127 weather stations in the study extent.

Over the study period, the median estimated daily rainfall per premises was 0.1 mm day^−1^ (IQR: 0 to 2.8 mm day^−1^, maximum = 106.5 mm day^−1^). No statistically significant associations were detected between time-lagged rainfall covariates and hazard of infection (Table 2). Moderate temporal correlation (*ρ*≈0.60) was observed between rainfall data 1 day apart, and between rainfall and relative humidity measurements conducted within 1 day of each other. A detailed correlation matrix of all continuous covariates is provided in Supporting Information S1.

**Table 2:** Univariable analysis of the association between meteorological covariates (time-changing and time-lagged) and time to infection of premises in the largest cluster (n = 3153), northwest of Sydney, during the 2007 equine influenza outbreak in Australia.

Meteorological Factor	Time-lag	*b*	SE(*b*)	LRT	*df*	*P*-value^a^
Rainfall (mm day^−1^)	*t_−1_*	0.006	0.033	0.0	1	0.852
	*t_−2_*	−0.005	0.028	0.0	1	0.870
	*t_−3_*	−0.045	0.037	1.5	1	0.215
	*t_−4_*	−0.024	0.027	0.9	1	0.342
	*t_−5_*	−0.028	0.031	0.9	1	0.344
Relative humidity (%),	*t_−1_*	*nonlinear spline*		29.9	4	<0.001
measured daily at 9 am	*t_−2_*	*nonlinear spline*		29.4	4	<0.001
	*t_−3_*	*nonlinear spline*		14.3	4	0.006
	*t_−4_*	*nonlinear spline*		47.1	4	<0.001
	*t_−5_*	*nonlinear spline*		41.9	4	<0.001
Relative humidity (%),	*t_−1_*	*nonlinear spline*		47.7	4	<0.001
measured daily at 3 pm	*t_−2_*	*nonlinear spline*		39.0	4	<0.001
	*t_−3_*	*nonlinear spline*		35.1	4	<0.001
	*t_−4_*	*nonlinear spline*		71.6	4	<0.001
	*t_−5_*	*nonlinear spline*		81.4	4	<0.001
Maximum daily air	*t_−1_*	*nonlinear spline*		58.4	4	<0.001
temperature (°C)	*t_−2_*	*nonlinear spline*		44.2	4	<0.001
	*t_−3_*	*nonlinear spline*		64.1	4	<0.001
	*t_−4_*	*nonlinear spline*		49.5	4	<0.001
	*t_−5_*	*nonlinear spline*		47.2	4	<0.001
Minimum daily air	*t_−1_*	−0.040	0.031	1.6	1	0.204
temperature (°C)	*t_−2_*	−0.033	0.032	1.1	1	0.289
	*t_−3_*	−0.068	0.032	4.6	1	0.031
	*t_−4_*	−0.052	0.033	2.5	1	0.111
	*t_−5_*	−0.027	0.032	0.7	1	0.395
Maximum daily wind	*t_−1_*	*nonlinear spline*		10.2	4	0.038
speed (km hour^−1^)	*t_−2_*	*nonlinear spline*		19.6	4	<0.001
	*t_−3_*	*nonlinear spline*		52.0	4	<0.001
*undirected* b	*t_−4_*	*nonlinear spline*		35.5	4	<0.001
	*t_−5_*	*nonlinear spline*		14.9	4	0.005

a *P*-values derived from likelihood ratio tests (LRT) comparing univariable to null Cox regression models.

b Maximum daily wind speed based on wind from all directions (‘undirected’), making no assumption concerning nearest infected premises assumption.

**Figure 5:**
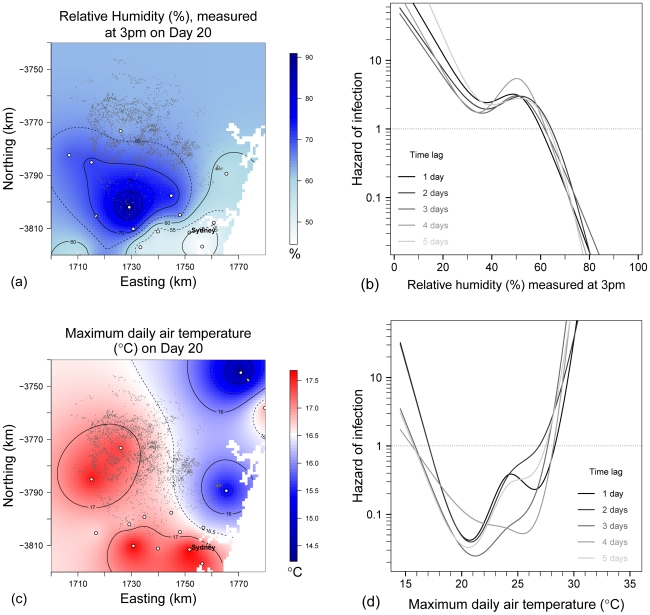
Smoothing of daily meteorological data and estimation of the association with premises-level hazard of infection in the largest cluster of the 2007 outbreak of equine influenza in Australia. Daily meteorological data provided by Australian Bureau of Meteorology weather stations (white closed circles) were smoothed using kriging, and time-lagged by 1–5 days. (a) Smoothed estimate of relative humidity measured at 3 pm on Day 20 of the outbreak. Small grey dots denote the horse premises. (b) Restricted cubic splines of the crude relationship between hazard of infection and relative humidity (3 pm measurement) at time-lags of 1–5 days over the entire study period. (c) Smoothed daily maximum air temperature on Day 20 and (d) the relationship between daily maximum air temperature and hazard of infection, by time lag.

Relative humidity data measured twice daily (at 9 am and 3 pm) were available from eighteen weather stations (Figure 5a). The mean of the estimated 9 am and 3 pm relative humidity measurements for the horse premises under observation were 70.8% (SD = 17.5%) and 52.9% (SD = 20.3%), respectively. Conditions were drier when measured at the same station at 3 pm compared to 9 am, on any given day, with paired relative humidity measurements 16.0% on average lower in the afternoon (95% CI: 15.3, 16.8%). Moderate to high temporal autocorrelation (*ρ*≈0.70) was observed between 9 am and 3 pm relative humidity data on the same day and at the same time 1 day apart.

A negative cubic relationship was observed between relative humidity and hazard of infection (Figure 5b). Risk of equine influenza infection was highest in dry conditions (<20% relative humidity), decayed rapidly until increasing at intermediate relative humidity (40–60%). Once relative humidity was >80% there was effectively no risk. This relationship was independent of whether relative humidity was measured at 9 am or 3 pm, and was also independent of the time lag applied (Table 2). The strongest statistical association was with the 3 pm measurement time-lagged by 5 days, thus, ‘RH_3PM *t_−5_*’ was selected as a proxy for relative humidity, irrespective of diurnal variation or time-lag.

Daily surface air temperature data were available from 21 weather stations (Figure 5c). The mean of the estimated daily maximum and minimum temperatures at the 3153 horse premises was 24.0°C (95% CI: 22.4, 25.7), and 12.6°C (95% CI: 9.1, 16.1°C), respectively. There was an increasing trend in temperature across the study period as the season changed from spring to summer, and a low level of correlation (*ρ*≈0.40) between maximum and minimum temperature measured on the same day. Minimum daily temperature data 1–4 days apart were moderately to highly correlated (0.61≤*ρ*≤0.75), less correlation was observed between maximum daily temperatures 1 day apart (*ρ*≈0.53), and a low cross-correlation (0.30<*ρ*<0.50) was observed between minimum daily temperature, rainfall and 9 am relative humidity data for 1–5 days.

A highly nonlinear relationship was observed between infection and maximum daily air temperature (Figure 5d), with risk of infection greatest toward both extremes of the range of observed maximum temperatures (<16°C and >28°C). The statistical strength of this association was greatest at a time-lag of 3 days (Table 2), however, the shape was consistent across time-lags. Hazard of infection increased linearly as minimum daily temperatures decreased, and the statistical strength of this association was also greatest when a time-lag of 3 days was applied. Combining daily maximum and minimum measurements into a midpoint daily temperature resulted in weaker associations (*data not shown*).

Hourly wind velocity data were available from sixteen weather stations (Figure 2b). Wind conditions varied considerably in time with little temporal autocorrelation observed (see correlation matrix in Supporting Information S1). There was no clearly discernible predominant wind pattern over the study period. The median of the maximum daily reported wind speeds estimated for each premises (*from all directions*) was 26.6 km hour^−1^ (IQR: 22.3, 33.3 km hour^−1^, maximum = 73.3 km hour^−1^).

**Figure 6:**
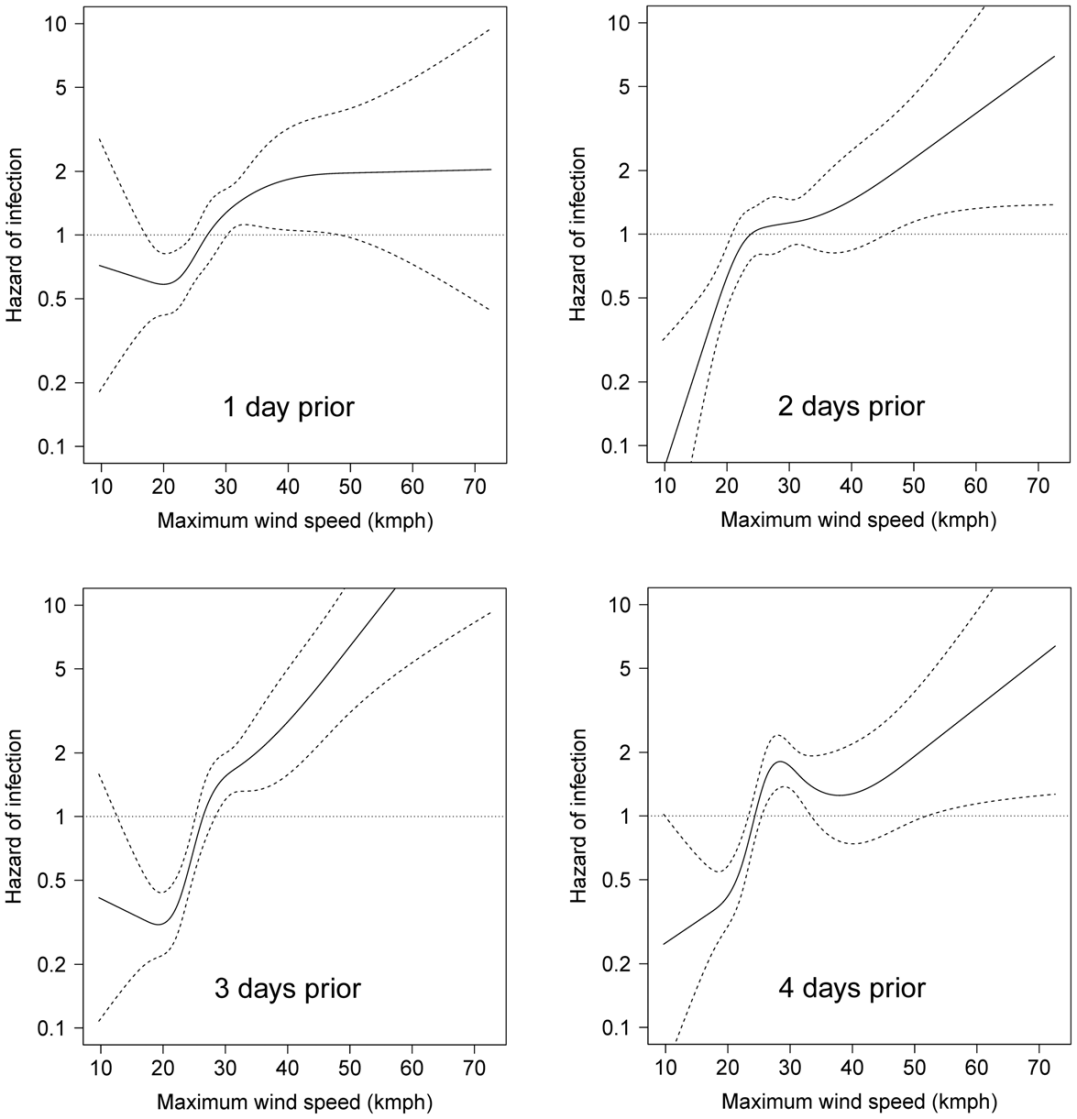
The crude relationship between hazard of infection and maximum daily wind speed, *from all directions*, in the largest cluster of the 2007 outbreak of equine influenza outbreak, by time lag. Estimates are based on hourly wind data from all directions and time-lagged by 1–4 days. Dashed lines represent 95% confidence intervals.

**Figure 7:**
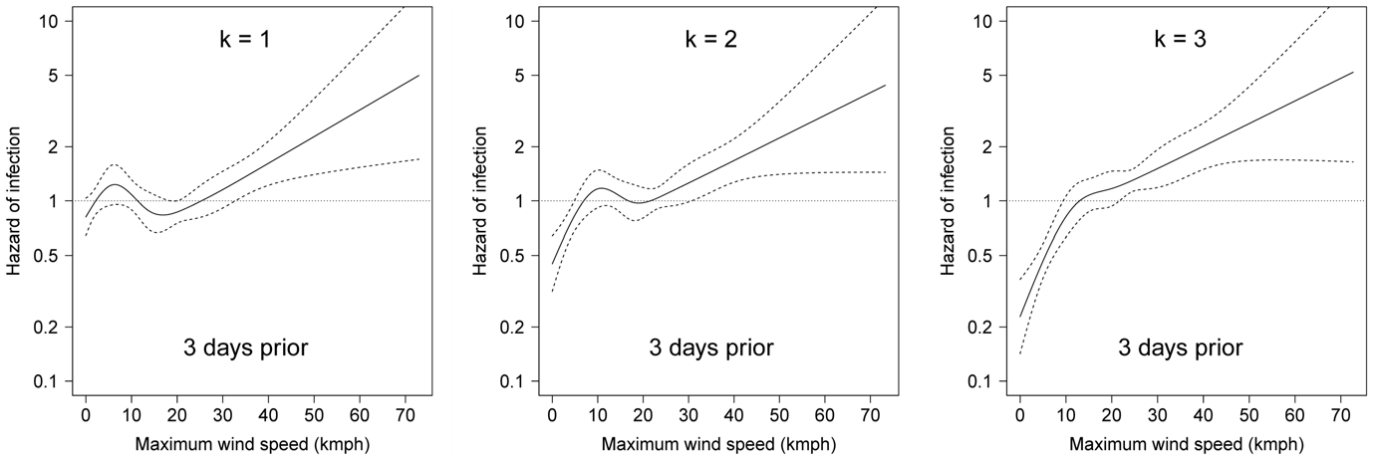
The crude relationship between hazard of infection and maximum daily wind speed selected from the direction of the *k* nearest infected premises (time-lagged by 3 days) in the largest cluster of the 2007 outbreak of equine influenza in Australia. Estimates are based only on hourly wind data from within 45° arcs centred on the direction of the *k* nearest infected premises, for *k* = 1,2,3. Arcs may overlap if nearest *k* infected premises are in the same direction (see Figure 2c for details). Dashed lines represent 95% confidence intervals.

The univariate relationship between hazard of infection and wind speed, making no directional assumptions (’undirected’), is presented in Figure 6, by time-lag. Maximum daily wind speed, lagged by 3 days, had the strongest statistical association with the outcome (Table 2). Increased hazard of infection was observed on days when the maximum daily wind speed was >30 km hour^−1^. The univariate relationships between hazard of infection and maximum daily wind speed from the direction of the *k* nearest neighbours are presented in Table 3, and plots of the restricted cubic splines of these relationships are shown in Figure 7 (only for a time-lag of 3 days). The strongest statistical association between any wind speed covariate and hazard of infection was identified based on ‘directed’ wind speed from the direction of the three nearest neighbours, time-lagged by 3 days.

**Table 3:** Univariable analysis of the association between directed wind speed covariates (time-changing and time-lagged) and time to infection of premises in the largest cluster (n = 3153), northwest of Sydney, during the 2007 equine influenza outbreak in Australia.

Meteorological Factor	Time-lag	Term	LRT	*df*	*P*-value^b^
Maximum daily wind	*t_−1_*	*nonlinear spline*	3.8	4	0.430
speed (km hour^−1^)	*t_−2_*	*nonlinear spline*	9.1	4	0.058
	*t_−3_*	*nonlinear spline*	16.5	4	0.002
*directed* (*k* = 1)^a^	*t_−4_*	*nonlinear spline*	6.6	4	0.159
	*t_−5_*	*nonlinear spline*	3.4	4	0.499
Maximum daily wind	*t_−1_*	*nonlinear spline*	14.0	4	0.007
speed (km hour^−1^)	*t_−2_*	*nonlinear spline*	25.3	4	<0.001
	*t_−3_*	*nonlinear spline*	34.5	4	<0.001
*directed* (*k* = 2)^a^	*t_−4_*	*nonlinear spline*	8.2	4	0.083
	*t_−5_*	*nonlinear spline*	24.6	4	<0.001
Maximum daily wind	*t_−1_*	*nonlinear spline*	41.2	4	<0.001
speed (km hour^−1^)	*t_−2_*	*nonlinear spline*	49.5	4	<0.001
	*t_−3_*	*nonlinear spline*	75.6	4	<0.001
*directed* (*k* = 3)^a^	*t_−4_*	*nonlinear spline*	38.0	4	<0.001
	*t_−5_*	*nonlinear spline*	52.3	4	<0.001

a Maximum daily wind speed (‘directed’) based on wind only from within 45° arcs centred on the direction of the *k* nearest infected premises for *k* = 1,2,3 (see Figure 2 for details) assuming that premises were infectious for 14 days and one of the nearest *k* infective premises was the source of infection.

b *P*-values derived from likelihood ratio tests (LRT) comparing univariable to null Cox regression models.

The following five candidate meteorological variables were consequently selected for multivariable analysis: linear terms for rainfall and minimum daily air temperature, both time-lagged by 3 days, a restricted cubic spline for relative humidity measured at 3 pm time-lagged by 5 days, and splines of maximum daily air temperature and maximum daily wind speed from the direction of the three nearest infected premises, both time-lagged by 3 days.

#### Horse premises attributes and hazard of infection

**Figure 8:**
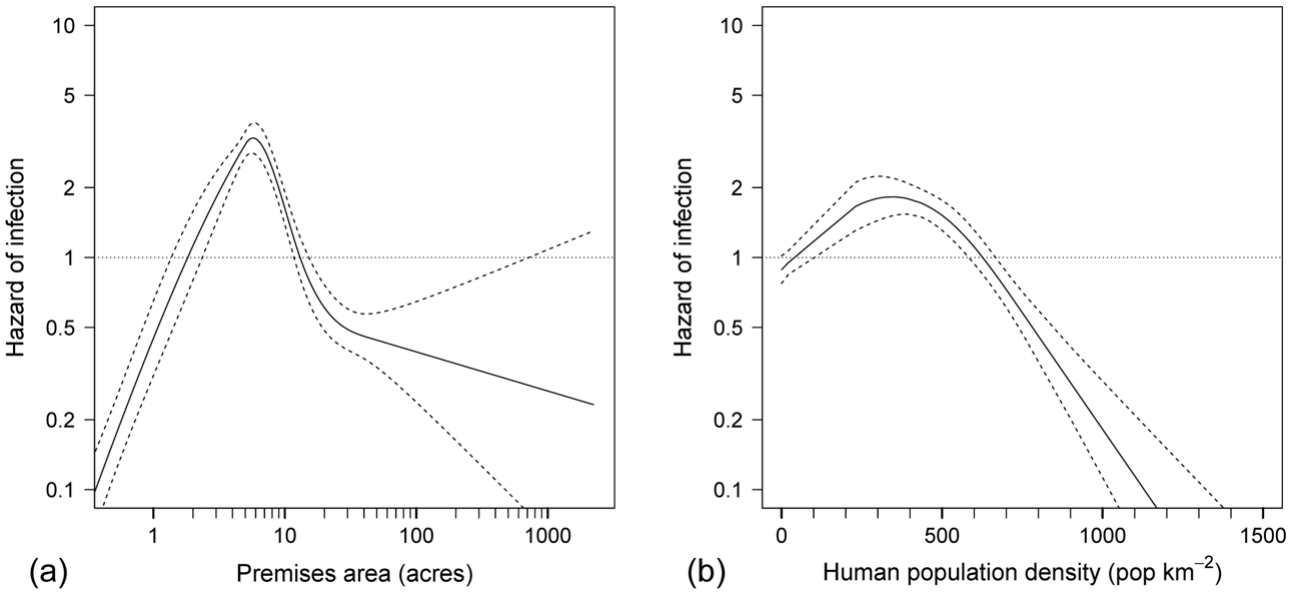
Crude nonlinear relationships between hazard of infection and non-meteorological covariates in the largest cluster of the 2007 outbreak of equine influenza in Australia. (a) The relationship between hazard of infection and premises area, and (b) the relationship between hazard of infection and local human population density (people residing within approximately 1 km of the horse premises). Dashed lines represent 95% confidence intervals.

Horse premises in the Northwest Sydney cluster were highly skewed in terms of their land area and the number of horses they held at the time of the outbreak. The median premises held 2 horses (IQR: 1, 5 horses; maximum: 139 horses) on 5.1 acres (IQR: 4.8, 15.2 acres; maximum: 2 225 acres). These variables were log transformed for all further analyses, with results back-transformed for presentation. Highly non-linear crude relationships were observed between hazard of infection and premises area and horse density (Table 4 and Figure 8a). Medium sized (4.8–15.2 acres) and medium density premises (1–5 acres per horse) were at increased risk of infection, as were horse premises that shared a fence with another horse premises. Hazard also increased with the number of horses held on a premises; this trend was well represented by categorisation based on quartiles.

**Table 4:** Univariable analysis of non-meteorological covariates with time to infection of premises in the largest cluster (n = 3153), northwest of Sydney, during the 2007 equine influenza outbreak in Australia.

Factor	Category	No.	Hazard ratio	(95% CI)	*P*-value^a^
***Premises attributes***					
Area (acres)	>15.2	788	0.99	(0.85, 1.15)	<0.001
	5.1–15.2	788	1.94	(1.69, 2.23)	
	4.8–5.1	789	2.09	(1.83, 2.40)	
	<4.8	788	1.00		
Horse density	>1.00	776	1.50	(1.29, 1.74)	<0.001
(horses acre^−1^)	0.40–1.00	799	2.51	(2.18, 2.89)	
	0.20–0.40	787	1.85	(1.63, 2.17)	
	<0.20	791	1.00		
Number of horses	>5	662	3.28	(2.82, 3.82)	<0.001
	3–5	902	2.48	(2.14, 2.88)	
	2	787	2.08	(1.79, 2.43)	
	1	802	1.00		
Length of shared fence	>300	742	1.45	(1.29, 1.63)	<0.001
with other horse	1–300	725	1.64	(1.47, 1.84)	
premises (m)	0	1686	1.00		
Vaccination status^b^	Yes	490	0.28	(0.04, 2.13)	0.137
	No	2663	1.00		
***Spatial covariates***					
Elevation (m)	>115	785	0.72	(0.63, 0.82)	<0.001
	45–115	777	0.66	(0.58, 0.76)	
	25–45	786	1.02	(0.90, 1.15)	
	<25	805	1.00		
Human population	>500	1059	1.05	(0.94, 1.18)	<0.001
density (people km^−2^)	1–500	954	1.29	(1.48, 1.44)	
	0	1140	1.00		
Distance to nearest	>2.2	787	1.23	(1.08, 1.41)	0.021
main road (km)	1.1–2.2	789	1.14	(1.00, 1.31)	
	0.4–1.0	788	1.11	(0.97, 1.27)	
	<0.4	786	1.00		

a *P*-values derived from likelihood ratio (LRT) tests comparing univariable to null Cox regression models.

b Time-changing covariate.

A trend existed across the study area in terms of premises elevation and surrounding human population density. Hazard of infection was higher on horses premises located at lower elevations (<45 m) and >2.2 km from main roads (Table 4). Risk was also higher on horse premises located in peri-urban areas (human population densities between 1–500 people km^−2^) compared to premises located either away from residential areas (human population density within 1 km = 0) or within urban areas (>500 people km^−2^) (Figure 8b).

Premises area and premises horse density were the only highly correlated pairing (*ρ* = −0.74), amongst the premises attribute variables. Of these two covariates, premises area was the more strongly associated with the outcome. The following premises attribute variables were therefore included in multivariable analysis: splines of premises area and local human population density, number of horses, length of shared fence with other horse premises, premises elevation and distance to the nearest main road. Vaccination status was retained as it was considered an *a priori* confounder.

### Multivariable analysis

The final model is presented in Table 5. Two variables were eliminated during multivariable model-building: ‘distance to nearest main road’ and ‘minimum daily air temperature’. No first order interaction terms were significant at *P*<0.05.

**Table 5:** Final multivariable Cox regression model for time to infection of premises in the largest cluster (n = 3153), northwest of Sydney, during the 2007 equine influenza outbreak in Australia.

Factor	Category	Hazard ratio	(95% CI)	*P*-value^a^
***Meteorological covariates***				
Rainfall (mm day^−1^), *t_−_*_***3***_ b	*Linear*	0.91	(0.82, 1.00)	0.055
Relative humidity (%),	*Nonlinear spline*	—	—	<0.001
measured daily at 3pm, *t_−_*_***5***_ b				
Maximum daily air	*Nonlinear spline*	—	—	<0.001
temperature (°C), *t_−3_* b				
Maximum daily wind speed,	*Nonlinear spline*	—	—	<0.001
(km hour^−1^), *t _−3_* b				
*directed* (*k* = 3)^c^				
***Premises attributes***				
Area (acres)	*Nonlinear spline*	—	—	<0.001
Number of horses	>5	3.16	(2.70, 3.69)	<0.001
	3–5	2.19	(1.89, 2.55)	
	2	1.93	(1.66, 2.26)	
	1	1.00		
Length of shared fence	>300	1.30	(1.15, 1.48)	<0.001
with other horse premises (m)	1–300	1.27	(1.13, 1.43)	
	0	1.00		
Vaccination status^b^	Yes	0.28	(0.04, 2.09)	0.134
	No	1.00		
***Spatial covariates***				
log_10_(Elevation (m))	*Linear*	0.58	(0.51, 0.67)	<0.001
Human population density	*Nonlinear spline*	—	—	<0.001
(people km^−2^)				

Number of events = 1727; Log likelihood = −12,847.4; *df* = 25; *P*<0.001; R^2^ = 25.8%.

a *P*-values derived from Likelihood ratio tests (LRT).

b Time-changing covariate, time-lagged 3 or 5 days as noted.

c Maximum daily wind speed (‘directed’) based on wind only from within 45° arcs centred on the direction of the three nearest infected premises assuming that premises were infectious for 14 days and one of the three nearest infective premises was the source of infection.

The shape of the restricted cubic splines representing the nonlinear relationships between hazard of infection and relative humidity, maximum daily air temperature, maximum daily wind speed (from the direction of the nearest three infected premises), premises area and human population density, were all largely unchanged from their crude forms (as presented in Figures 5, 6, 7, 8). Post-adjustment, rainfall was detected to be weakly protective. The increased hazard amongst premises with higher numbers of horses persisted, as did the reduction in hazard amongst premises at higher elevations, with a 42% reduction in risk for every order of magnitude increase in elevation. Premises that were adjacent to another horse premises were at increased hazard of equine influenza infection.

#### Model goodness-of-fit and residual analysis

The final model accounted for a quarter of the variability in the data (Schemper and Stare pseudo-R^2^ = 25.8%). No issues were identified based on inspection of martingale and deviance residuals, both overall, and when plotted against each variable included in the final model. Residual spatial structure was not evident in the empirical semivariogram of the deviance residuals, suggesting that spatial correlation was not unduly influencing our effect estimates (or their associated standard errors). Influence statistics identified only one important outlying premises, infected 36 days after the vaccination of the 2 horses on the property. These horses did not receive a second vaccination, whilst up to three doses may be required to attain protective immunity.

## Discussion

To our knowledge, this empirical analysis provides the first estimates of the contribution of humidity, air temperature and wind to the spread of an actual outbreak of influenza (‘in the field’). We have demonstrated that it is possible to detect an association between wind velocity and disease spread, and directly estimate the strength of such an association. This advances our understanding of the windborne spread of influenza from purely circumstantial association to a hypothesis statistically-tested with empirical data.

### Relative humidity and influenza spread

Our analysis shows that influenza spread in this cluster was highly dependent on relative humidity. Recent reviews [4], [5], [61] present contradictory results from laboratory trials of influenza A virus survival at intermediate humidities [62], [63], and disagreement concerning the importance of aerosol transmission. The negative cubic relationship that we observed between hazard of infection and relative humidity provides field validation for some of these laboratory trials. The curve in Figure 5b across the whole range of relative humidities observed under natural conditions, exactly complements the results presented by Hemmes et. al. [62] of inactivation of aerosolised influenza A virus under controlled conditions. Our findings also support the theory presented by Lowen et al. [6] that the relationship between influenza transmission and relative humidity is mediated by both virion and aerosol droplet nuclei stability. In cool dry conditions, droplets are desiccated and remain small, which may stabilise influenza aerosols and facilitate longer range transmission, whereas at high relative humidity, the droplets absorb water and settle [61]. The small rise in hazard of infection at intermediate relative humidities (40–60%) is perhaps due to a summation of two effects: as relative humidity increases within this range so too does viral survival [63], whilst droplet nuclei settle more readily. This rise was most pronounced in the spline of relative humidity time-lagged by 3 days, yet the 5 day time-lagged variable was the predictor in the group with the strongest statistical association with hazard of infection. Amongst all other groupings of autocorrelated meteorological variables, a time-lag of 3 days was the predictor with the strongest statistical association with hazard of infection, corresponding closely with the typical 1–3 day incubation period of equine influenza.

Recent research has suggested that in certain situations absolute humidity may better represent the relationship between air humidity and influenza A virus survival [64] and aerosol transmission [65]. However, the dependency is perhaps more complex [8], because the amount of water vapour that air can hold increases with temperature. Absolute and relative humidity are related metrics for the amount of water vapour in moist air. Absolute humidity is the mass of water vapour per cubic meter of total moist air, whereas, relative humidity is absolute humidity expressed as a percentage of the amount of water vapour needed for saturation at a specific temperature. We used relative humidity rather than absolute humidity because: the relative humidity data were more complete over the study period, corresponding 9 am and 3 pm air temperature data were not available for all data points so back-transformation of relative humidity measurements into absolute humidity would have resulted in less complete data, and we wanted to ensure we could directly compare our results with the original research describing the dependency between relative humidity, air temperature and influenza virus transmission and survival [6], [62].

### Air temperature and influenza spread

The shape of the highly nonlinear relationship that we observed between hazard of equine influenza infection and maximum daily air temperature suggests two mechanisms of influenza transmission. Hazard was lowest on days when the maximum air temperature was between 20–25°C, and greatly increased on days with lower and higher maximum temperatures. Aerosol transmission of influenza A viruses has been shown to be enhanced in cooler conditions [6], and on days when maximum daily temperature was <20°C the air temperature would be expected to remain in the optimal range for aerosol transmission equine influenza for longer. The marked increase in hazard of infection when maximum temperature was >25°C is also consistent with recent research. Whilst high temperatures block aerosol transmission of influenza A viruses, the success of animal-to-animal contact transmission increases at high temperatures [10] perhaps explaining the spread of influenza in warm tropical environments.

### Wind velocity and influenza spread

There is some consensus in the literature that airborne transmission of influenza is at least possible; however, there is strong disagreement about its importance [5]. In the cluster investigated, we observed an association between hazard of infection and increasing wind speed from the direction of nearby potential sources of equine influenza infection. A similar association was found when wind speed covariates were generated without making any directional assumptions, in effect testing the general hypothesis that hazard of infection was increased on days with increased wind speed (*from any direction*). Irrespective of our approach, wind speeds >30 km hour^−1^, lagged by 3 days, were consistently associated with increased hazard of infection.

In developing proxy covariates for the directed formulation of the daily wind speed covariates (‘WIND_SPD_dir(*k*)_’) certain assumptions were required. Wind data was only included if it was within a 45° arc of the nearest *k* infected premises to each uninfected premises on each observation day. The nearest *k* infected premises were assumed to be the only windborne source of equine influenza virus for a susceptible premises, and we assumed the duration of infectivity at the premises level was 14 days for all premises. The statistical strength of association between hazard of infection and wind speed increased as more nearest neighbours were cumulatively incorporated into the method of wind covariate generation, suggesting that the nearest neighbour was not always the only source of windborne infection. It would be computationally intensive to continue incorporating further nearest neighbours into this method, so we cannot definitively state how far to extend this process. There must exist a point after which adding infected neighbours to the process of generating wind speed covariates results in weaker associations, as the associations were statistically stronger when wind speed covariates were generated with three nearest neighbours than when we made no directional assumptions. We can state that incorporating three nearest infected neighbours is better than only one or two, that our findings are relatively robust to the assumptions that we made whilst generating directed wind speed covariates, and that the detected association between hazard of infection and wind speed appears related to the direction of proximate infected premises.

The associations that we have detected between increasing wind speed and hazard of infection need to be interpreted in the context of our study design. There is potential for ecological fallacy in aggregated data analyses such as this, in which the unit of interest is not an individual animal but a group. Furthermore, it is not possible in such observational epidemiological analyses to definitively identify windborne spread from any other transmission route (direct contact, cough droplet and spread on fomites). Nonetheless, the detected association, presumably representing windborne spread of equine influenza, is biologically plausible, and its increasing strength with increasing wind speed from the direction of nearby infected premises is difficult to explain by spread through other means alone.

At wind speeds of >30 km hour^−1^ an aerosol of influenza droplet nuclei would only need to be stable for minutes to be able to infect horses on nearby premises. Equine influenza viruses have been shown to survive for periods of hours to days in soil and water, even in direct sunlight [12], and infected horses shed large amounts of virus (>10^3^ EID_50_/ml, 50% egg infective dose per ml of swab extract) throughout the roughly 7 days that they are infectious [66]. Infection is more reliably achieved by inhalation of aerosolised virus that intranasal inoculation [26], with a minimum infective dose of 10^2^ EID_50_/ml. We therefore consider it plausible that infected horses on one premises could cough or otherwise produce a sufficient quantity of aerosolised equine influenza virus, which after travelling wind-assisted could constitute an infectious dose for a horse on a nearby premises (whether inhaled immediately or after surviving a short period on soil or in drinking water).

A recent time-series analysis investigated correlation between the frequency of paediatric influenza A hospital admissions and several meteorological variables including wind velocity [15]. A statistically significant univariable association was observed between increasing wind velocity and increased influenza A hospital admissions, in data collected from one hospital and one weather station [15]. However, in multivariable analyses no association was observed between wind velocity and influenza A hospitalisations, perhaps due to the level of spatial and temporal data aggregation (across the hospital catchment and into 14-day time intervals). Aggregated analyses of the association of meteorological factors with the spread of the severe acute respiratory syndrome (SARS) in Beijing [16], and hand, foot and mouth disease (HFMD) of humans in Hong Kong [67], have found statistically significant associations with increasing wind velocity, albeit at much lower wind velocities. Atmospheric dispersal modelling of the picornavirus that causes foot and mouth disease (FMD) in cloven-hoofed ungulates has consistently found that the virus is likely to be dispersed even in calm conditions [17], [68], [69]. The dependency of influenza A virus survival on relative humidity [6], [62] is completely different to that of poliovirus [62], HFMD [70] and FMD virus [68] (all much smaller RNA viruses of the family *Picornaviridae*). Therefore, it is perhaps not unexpected that the survival of aerosols of influenza A viruses and picornaviruses could depend on different wind conditions.

When interpolating meteorological covariates and estimating nearest neighbour distances we used centroids to reduce the complexity of the analytical methods. For >99% of the premises in our dataset we estimate that the maximum distance between the centroid and premises boundary was <500 m. When the largest 1% of premises (in area) were excluded from the final model, the only regression coefficients to change by >20% were the two highest order spline components for relative humidity and maximum daily temperature, and these changes were not discernible in post-adjustment plots. We therefore consider our findings to be insensitive to measurement bias introduced by representing premises by their centroids.

Environmental variables capable of influencing airborne disease spread (such as local horse density, tree density or terrain undulation) vary considerably in the different regions and clusters of premises infected during the 2007 equine influenza outbreak in Australia. A potential limitation of this analysis was that we focussed on only one cluster (the largest and most dense cluster in terms of population at risk) from a very large outbreak. There were two considered reasons for our detailed focus: counting process survival analysis involves analysing a very large dataset (204,909 observations on 3153 premises); and owing to a wide variance in local environmental characteristics and potential for differences in disease transmission dynamics, mixing clusters in the same analysis might dilute any meaningful results. Before generalising our findings to the whole outbreak, or indeed other outbreaks, follow-up research to assess the importance of the risk factors investigated in broadly dissimilar environments, is therefore required.

A classical geostatistical approach [49] (kriging based on a least squares fit of an empirical variogram) was applied to interpolate premises-level meteorological covariates from weather station data. A more sophisticated model-based geostatistical approach [71] (maximum-likelihood based model fitting that does not rely on an empirical variogram) may be more appropriate. However, we considered that the numerous fine adjustments required to undertake a model-based approach to be impractical for fitting the 5616 separate models (117 days×24 hours×2 wind component vectors) that were required to produce hourly wind vector estimates at each of the 3153 individual premises locations across the entire study period. It was also not possible to assess the assumptions of stationarity or isotropy for each of the thousands of semivariogram models required to generate all of the daily meteorological covariates for each premises. These assumptions appeared justified based on semivariograms of the mean conditions for each meteorological covariate over the entire study period. Our interpolation approach could have been refined by incorporating elevation (regression kriging), a spatial trend or even anisotropy into the method. The study extent covered the Northern half of the Sydney basin, which is relatively flat, and bounded by a plateau of national parks where horses are prohibited. These refinements would be recommended when conducting further similar research of clusters located in more varied terrain.

In the cluster of infection investigated, disease did not appear to spread predominantly in any single direction. We purposefully focussed on this cluster rather than other large clusters in which a single global direction of spread has been noted [13], with the intention of estimating the typical contribution of wind to disease spread rather than circumstantially associating prevailing wind with the global direction of disease spread. In any cluster in which an overall direction of spread is detected, an important further research question remains: What proportion of this anisotropic spread is directly attributable to windborne disease spread? Our methods provide a means to answer this research question, and to retrospectively investigate the contribution of windborne aerosol spread to local disease spread during outbreaks such as the foot-and-mouth disease outbreak in the United Kingdom in 2001.

By restricting this analysis to a study period after the horse movement ban was put in place, we focussed this study on factors influencing the local spread of equine influenza. We also adjusted for a number of relevant confounders of the meteorological associations we aimed to estimate: vaccination status of horses on the premises, premises size (in terms of area and number of horses), whether premises were adjacent to another premises holding horses, and local human population density. A small misclassification bias is known to be present in the equine influenza dataset, due to under-reporting of infected premises by owners either attempting to avoid movement restrictions or who failed to detect infection [72]. A previous analysis found <1% under-reporting occurred in this region, suggesting that <13 infected premises were misclassified as uninfected [72]; we considered this bias negligible.

In conclusion, by combining influenza outbreak and concurrent meteorological data, we have shown how relative humidity, air temperature and wind velocity combined to influence the spread of an actual influenza outbreak. Hazard of equine influenza infection was higher when relative humidity was <60% and lowest on days when daily maximum air temperature was 20–25°C. Wind speeds >30 km hour^−1^ from the direction of nearby infected premises were associated with increased hazard of infection. Our analysis supports, and extends, the findings of studies into influenza A transmission conducted under controlled conditions. The relationships described are of direct importance for managing disease risk during influenza outbreaks in horses, and more generally, advance our understanding of the transmission of influenza A viruses under natural conditions.

## Supporting Information

Supporting Information S1 Survival analysis dataset formulation examples and correlations between explanatory variables in Cox regression modelling of factors associated with time to infection in the largest cluster of the 2007 outbreak of equine influenza in Australia.
(DOCX)
