# Supplementary material for: The Influence of Meteorology on the Spread of Influenza: Survival Analysis of an Equine Influenza (A/H3N8) Outbreak
Source: PLoS One. 2012 Apr 20;7(4):e35284. doi: 10.1371/journal.pone.0035284 (PMC3335077; doi:10.1371/journal.pone.0035284)
Supplement: Supporting Information S1 — Survival analysis dataset formulation examples and correlations between explanatory variables in Cox regression modelling of factors associated with time to infection in the largest cluster of the 2007 outbreak of equine influenza in Australia. (DOCX) [file pone.0035284.s001.docx]

**Table S1: Example time-independent survival analysis dataset formulation for an infected premises, in Cox regression modelling of factors associated with time to infection in the largest cluster of the 2007 outbreak of equine influenza in Australia.**

| **INFECTED PREMISES (unvaccinated)** | | | | | | | | | | | |
| --- | --- | --- | --- | --- | --- | --- | --- | --- | --- | --- | --- |
| **ID** | **IP** | **DAY** | **VACC** | **VACC_DAYS** | **HORSE_N** | **AREA** | **CENT_DIST** | **CENT_DIR** | **ROAD_DIST** | **ELEV** | **WIND_ST** |
|  |  |  |  |  |  |  |  |  |  |  |  |
| 0001 | 1 | 55 | 0 | – | 11 | 84.3 | 21.0 | 36.0 | 0.6 | 26 | 8 |
|  |  |  |  |  |  |  |  |  |  |  |  |

**Table S2: Example time-dependent (‘counting process’) survival analysis dataset formulation for an infected premises, in Cox regression modelling of factors associated with time to infection in the largest cluster of the 2007 outbreak of equine influenza in Australia.**

| **INFECTED PREMISES (unvaccinated)** | | | | | | | | | | | | | | |
| --- | --- | --- | --- | --- | --- | --- | --- | --- | --- | --- | --- | --- | --- | --- |
| **ID** | **START** | **STOP** | **IP** | **VACC_TD** | **HORSE_N** | **AREA** | **IP_DIST** | **IP_DIR** | **WIND_SPD (KMPH)** | | | **TEMP_MIN (°C)** | | |
|  |  |  |  |  |  |  |  |  | ***t_-1_*** | ***t_-2_*** | ***t_-3_*** | ***t_-1_*** | ***t_-2_*** | ***t_-3_*** |
|  |  |  |  |  |  |  |  |  |  |  |  |  |  |  |
| 0001 | 14 | 15 | 0 | 0 | 11 | 84.3 | 8.45 | 102.4 | 0 | 17 | 0 | 13.4 | 10.9 | 9.1 |
| 0001 | 15 | 16 | 0 | 0 | 11 | 84.3 | 8.45 | 102.4 | 8 | 0 | 17 | 14.1 | 13.4 | 10.9 |
| 0001 | 16 | 17 | 0 | 0 | 11 | 84.3 | 7.74 | 77.9 | 17 | 8 | 0 | 8.7 | 14.1 | 13.4 |
| 0001 | 17 | 18 | 0 | 0 | 11 | 84.3 | 7.74 | 77.9 | 11 | 17 | 8 | 12.2 | 8.7 | 14.1 |
| 0001 | 18 | 19 | 0 | 0 | 11 | 84.3 | 7.74 | 77.9 | 15 | 11 | 17 | 12.6 | 12.2 | 8.7 |
| …. | …. | …. | …. | …. | …. | …. | …. | …. | …. | …. | …. | …. | …. | …. |
| 0001 | 50 | 51 | 0 | 0 | 11 | 84.3 | 0.18 | 299.1 | 9 | 17 | 46 | 8.0 | 13.1 | 10.9 |
| 0001 | 51 | 52 | 0 | 0 | 11 | 84.3 | 0.18 | 299.1 | 31 | 9 | 17 | 11.0 | 8.0 | 13.1 |
| 0001 | 52 | 53 | 0 | 0 | 11 | 84.3 | 0.18 | 299.1 | 21 | 31 | 9 | 12.6 | 11.0 | 8.0 |
| 0001 | 53 | 54 | 0 | 0 | 11 | 84.3 | 0.18 | 299.1 | 0 | 21 | 31 | 12.4 | 12.6 | 11.0 |
| 0001 | 54 | 55 | 1 | 0 | 11 | 84.3 | 0.18 | 299.1 | 5 | 0 | 21 | 11.6 | 12.4 | 12.6 |
|  |  |  |  |  |  |  |  |  |  |  |  |  |  |  |

IP = premises infection status, DAY = date of onset, VACC = vaccination status (Yes =1), VACC_DAY = day of vaccination, VACC_TD = vaccination status (time-dependent variable), HORSE_N = number of horses, CENT_DIST/DIR = distance (km) and direction (degrees) from cluster centre, IP_DIST/DIR = distance (km) and direction (degrees) from nearest potential source premises, ROAD_DIST = distance (km) from nearest main road, WIND_ST = ID of nearest weather station, WIND_SPD = time-lagged maximum wind speed from within 45 ° of the direction of the nearest infected premises, TEMP_MIN = time-lagged minimum surface air temperature.

**Table S3: Example time-independent survival analysis dataset formulation for an uninfected premises, in Cox regression modelling of factors associated with time to infection in the largest cluster of the 2007 outbreak of equine influenza in Australia.**

| **UNINFECTED PREMISES (vaccinated)** | | | | | | | | | | | |
| --- | --- | --- | --- | --- | --- | --- | --- | --- | --- | --- | --- |
| **ID** | **IP** | **DAY** | **VACC** | **VACC_DAYS** | **HORSE_N** | **AREA** | **CENT_DIST** | **CENT_DIR** | **ROAD_DIST** | **ELEV** | **WIND_ST** |
|  |  |  |  |  |  |  |  |  |  |  |  |
| 0002 | 0 | 131 | 1 | 77 | 2 | 41.5 | 21.5 | 79.0 | 1.8 | 199 | 8 |
|  |  |  |  |  |  |  |  |  |  |  |  |

**Table S4: Example time-dependent (‘counting process’) survival analysis dataset formulation for an uninfected premises, in Cox regression modelling of factors associated with time to infection in the largest cluster of the 2007 outbreak of equine influenza in Australia.**

| **UNINFECTED PREMISES (vaccinated)** | | | | | | | | | | | | | | |
| --- | --- | --- | --- | --- | --- | --- | --- | --- | --- | --- | --- | --- | --- | --- |
| **ID** | **START** | **STOP** | **IP** | **VACC_TD** | **HORSE_N** | **AREA** | **IP_DIST** | **IP_DIR** | **WIND_SPD (KMPH)** | | | **TEMP_MIN (°C)** | | |
|  |  |  |  |  |  |  |  |  | ***t_-1_*** | ***t_-2_*** | ***t_-3_*** | ***t_-1_*** | ***t_-2_*** | ***t_-3_*** |
|  |  |  |  |  |  |  |  |  |  |  |  |  |  |  |
| 0002 | 14 | 15 | 0 | 0 | 2 | 41.5 | 10.96 | 59.8 | 13 | 5 | 11 | 14.1 | 7.5 | 6.5 |
| 0002 | 15 | 16 | 0 | 0 | 2 | 41.5 | 10.96 | 59.8 | 0 | 13 | 5 | 10.7 | 14.1 | 7.5 |
| 0002 | 16 | 17 | 0 | 0 | 2 | 41.5 | 11.52 | 67.2 | 17 | 8 | 11 | 6.7 | 10.7 | 14.1 |
| 0002 | 17 | 18 | 0 | 0 | 2 | 41.5 | 10.39 | 65.5 | 13 | 17 | 8 | 13.2 | 6.7 | 10.7 |
| …. | …. | …. | …. | …. | …. | …. | …. | …. | …. | …. | …. | …. | …. | …. |
| 0002 | 75 | 76 | 0 | 0 | 2 | 41.5 | 5.44 | 111.5 | 21 | 0 | 0 | 11.0 | 19.0 | 13.2 |
| 0002 | 76 | 77 | 0 | 0 | 2 | 41.5 | 5.44 | 111.5 | 15 | 21 | 0 | 13.8 | 11.0 | 19.0 |
| 0002 | 77 | 78 | 0 | 1 | 2 | 41.5 | 5.44 | 111.5 | 22 | 15 | 21 | 14.7 | 13.8 | 11.0 |
| …. | …. | …. | …. | …. | …. | …. | …. | …. | …. | …. | …. | …. | …. | …. |
| 0002 | 128 | 129 | 0 | 1 | 2 | 41.5 | 3.85 | 70.6 | 18 | 24 | 8 | 22.1 | 19.4 | 17.8 |
| 0002 | 129 | 130 | 0 | 1 | 2 | 41.5 | 3.85 | 70.6 | 26 | 18 | 24 | 12.1 | 22.1 | 19.4 |
| 0002 | 130 | 131 | 0 | 1 | 2 | 41.5 | 30.01 | 97.7 | 18 | 26 | 0 | 16.0 | 12.1 | 22.1 |
|  |  |  |  |  |  |  |  |  |  |  |  |  |  |  |

IP = premises infection status, DAY = date of onset, VACC = vaccination status (Yes =1), VACC_DAY = day of vaccination, VACC_TD = vaccination status (time-dependent variable), HORSE_N = number of horses, CENT_DIST/DIR = distance (km) and direction (degrees) from cluster centre, IP_DIST/DIR = distance (km) and direction (degrees) from nearest potential source premises, ROAD_DIST = distance (km) from nearest main road, WIND_ST = ID of nearest weather station, WIND_SPD = time-lagged maximum wind speed from within 45 ° of the direction of the nearest infected premises, TEMP_MIN = time-lagged minimum surface air temperature.

**Table S5: Correlations between continuous explanatory variables analysed for associations with time to infection of premises in the largest cluster of the 2007 outbreak of equine influenza in Australia.**

AREA = premises area, HORSE_DENSITY = horses per acre, HORSE_NUMBER = number of horses, ELEV = premises elevation, HUMAN_DENS = population within 1 km of premises centroid, ROAD_DIST = distance from nearest main road, RAIN = rainfall, RH_9AM/RH_3PM = Relative humidity measured at 9am/3pm daily, TEMP_MAX/TEMP_MIN = maximum/minimum air temperature, WIND_SPD = maximum wind speed from within 45° of the direction of the nearest infected premises.
